# Finding Someone to Blame: The Link Between COVID-19 Conspiracy Beliefs, Prejudice, Support for Violence, and Other Negative Social Outcomes

**DOI:** 10.3389/fpsyg.2021.726076

**Published:** 2022-01-14

**Authors:** Jakub Šrol, Vladimíra Čavojová, Eva Ballová Mikušková

**Affiliations:** Institute of Experimental Psychology, Centre of Social and Psychological Sciences, Slovak Academy of Sciences, Bratislava, Slovakia

**Keywords:** conspiracy theories, COVID-19, prejudice, support for violence, negative social outcomes

## Abstract

One of the appeals of conspiracy theories in times of crises is that they provide someone to blame for what has happened. Thereby, they increase distrust, negative feelings, and hostility toward implicated actors, whether those are powerful social outgroups or one’s own government representatives. Two studies reported here examine associations of COVID-19 conspiracy theories with prejudice, support for violence, and other and negative social outcomes. In Study 1 (*N* = 501), the endorsement of the more specific conspiracy theories about the alleged role of China was associated with more prejudiced views of Chinese and Italian people. In Study 2 (*N* = 1024), lowered trust in government regulations and increased hostility associated with the COVID-19 and generic conspiracy beliefs were correlated with justification of and willingness to engage in non-compliance with regulations, violent attacks on 5G masts, and anti-government protests. Across both of the studies, higher exposure to news about COVID-19 was associated with lower endorsement of conspiracy theories, but also with increased feelings of anxiety and lack of control, which in turn were correlated with higher COVID-19 conspiracy beliefs endorsement. We highlight the potential social problems which are associated with the wide-spread endorsement of COVID-19 conspiracy theories.

## Introduction

Since the first reported cases of the new disease caused by SARS-CoV-2 virus at the end of the year 2019 rapidly grew into global pandemic, the spread of the disease was accompanied by a massive surge of conspiracy theories, that are known to arise in times of increased uncertainty, when people feel threatened and lacking control (van Prooijen and Douglas, 2017; Douglas et al., 2019; although see Stojanov and Halberstadt, 2020). And as the nature of the threat and uncertainty changed over the time, the specific content of conspiracy theories also mutated (for various categories and subcategories of conspiracy theories related to COVID-19 pandemic, see van Mulukom et al., 2020). For example, at the beginning of pandemic, conspiracy theories seemed to be focused mostly on the origin of the virus (whether it was created artificially, even as a bioweapon, and released intentionally, either by China or the United States) and on the dangerousness of the virus (it is a hoax, it is a flu). As it became apparent that vaccination will likely provide the best way to deal with the new virus, many countries saw the rise of conspiracy theories specifically related to vaccines against COVID-19. Later still, growing frustration with the strict health-preventive measures that had to be issued by many countries likely contributed to the rise of conspiracy beliefs that were focused mostly at the governmental measures issued to counter the pandemic (testing for COVID-19 as a means to collect your genetic material or inserting microchips into your brain, lockdown and vaccination as government means to control the masses). In Slovakia, where both of the present studies were conducted, conspiracy theories have become so widespread that a public poll conducted in October 2020 among Slovak middle-school teachers showed that almost one-third of them believe that COVID-19 vaccination means the population will be inserted with “nano-chips” in their body (Gdovinová, 2020).

All of this provided researchers a fertile ground to examine the role of various cognitive, personality and social predictors of beliefs in conspiracy theories (e.g., Biddlestone et al., 2020; Bruder and Kunert, 2020; De Coninck et al., 2021; Hornsey et al., 2021; Oleksy et al., 2021; Šrol et al., 2021). Other researchers focused on the consequences of conspiracy theory endorsement, such as increased reluctance to follow regulations for containing the pandemic (Erceg et al., 2020; Lazarević et al., 2021), vaccination hesitancy (Hornsey et al., 2020; Teovanović et al., 2020; Soveri et al., 2021), and spread of prejudice, xenophobia, extremism, and violence (Jolley and Paterson, 2020; Sorokowski et al., 2020; Levinsson et al., 2021). Building on these previous efforts, in this paper we aim to explore cognitive (news exposure) and emotional (anxiety, lack of control) correlates of the endorsement of conspiracy theories at two points of time in the pandemic, as well as the association between conspiracy beliefs and different negative social outcomes most salient at the beginning of the 1st wave vs. at the beginning of the 2nd wave of pandemic in Slovakia.

When examining conspiracy theories, first difficulty arises when trying to define what they are. Some authors stress that they refer to unfounded, unverified and officially unaccepted information that warn against dangerous plots of the powerful groups against the unsuspecting public (Panczová, 2017), while others focus on causal explanations of events by referring to secret plots and malicious intent (Uscinski, 2019). The first group highlights their epistemically suspect status (unverified and often unfalsifiable, rejected by epistemological authorities, probably false), the other focuses on the propensity to casually explain events by attributing malicious intent either to powerful in-groups or threatening out-groups. Before we introduce our research design in more detail, we first present brief description of emotional and cognitive predictors of conspiracy theories endorsement in times of crises and then we review the evidence for the link between conspiracy theory endorsement and prejudice of social outgroups, as well as justification of and willingness to engage in violent social actions.

### Anxiety, Lack of Control and Exposure to News About COVID-19 as Predictors of COVID-19 Conspiracy Beliefs

The rise of conspiracy theories in times of crises have been linked with feelings of anxiety and lack of control that prompts sense-making mechanisms, which, in turn, often lead to a higher susceptibility to various conspiracy explanations of the events taking place (van Prooijen and Douglas, 2017; Douglas et al., 2019; van Prooijen, 2019). In threatening situations people try to restore their sense of control and meaning to reduce anxiety, by several mechanisms, such as quick cognitive closure (Heine et al., 2006) or compensatory control (Brotherton, 2015). Several studies showed that strengthening people’s sense of control reduced beliefs in conspiracy theories (van Prooijen and Acker, 2015), while the threats to personal control and feelings of powerlessness increased the perception of patterns in random stimuli (Whitson and Galinsky, 2008), higher endorsement of popular specific conspiracy theories (Abalakina-Paap et al., 1999; van Prooijen and Acker, 2015), and higher generic conspiracy mentality (Bruder et al., 2013; but see Stojanov and Halberstadt, 2020). Associations between anxiety, lack of control and higher endorsement of conspiracy theories about COVID-19 was confirmed also by recent studies from the pandemic period (Biddlestone et al., 2020; Kim and Kim, 2021; Oleksy et al., 2021; Sallam et al., 2021; Šrol et al., 2021). On the other hand, COVID-19 pandemic affected people not only through increased feelings of anxiety, but as the pandemic unfolded, also the solutions to contain pandemic became the source of new conspiracy theories.

Endorsement of COVID-19 conspiracy theories has been linked also with increased social media exposure (Bridgman et al., 2020; Freeman et al., 2020; De Coninck et al., 2021), but only few examined how exposure to news about infected cases and death tolls in official sources affects prevalence of conspiracy theories (De Coninck et al., 2021). Moreover, increased exposure to news can have also indirect effect on conspiracy theories endorsement through the anxiety the information overload can cause (Eppler and Mengis, 2004). The link between increased media exposure and anxiety was shown also during the pandemic (Gao et al., 2020; Kwok et al., 2020; Xiong et al., 2020) and in this study we will examine whether it is further associated with endorsement of COVID-19 conspiracy theories.

### Negative Social Outcomes Associated With COVID-19 Conspiracy Beliefs

Conspiracy theories and prejudices are closely connected, because in the center of conspiracy beliefs is an attribution of malevolent intention, which serves both as an explanation of and finding meaning in complex social events and also finding the responsible culprit for the adverse events. Typically, in a modern society many prejudices remain hidden and discrimination subtle. However, anxiety, stressful life events and perceived lack of control can contribute to increased prejudice and attribution of perceived threats to outgroups and enemies (e.g., Sullivan et al., 2010; Fritsche et al., 2011). In such situations, adoption of conspiracy beliefs may serve the social motive of protecting one’s ingroup against the outgroup which is perceived as hostile (Douglas et al., 2017; van Prooijen, 2019).

Indeed, many studies have found an association between belief in conspiracy theories and prejudiced and discriminatory attitudes toward Jews (Golec de Zavala and Cichocka, 2012; Kofta et al., 2020), Muslims (Swami et al., 2018), European immigrants (Jolley et al., 2020), or Americans (Imhoff and Bruder, 2014). However, the specific content of a conspiracy theory may be important in this regard. A study by Oleksy et al. (2021) showed that only those COVID-19 conspiracy theories which feature a powerful outgroup that threatens one’s ingroup have led to negative feelings toward Chinese and Italians (two outgroups strongly associated with the first wave of the COVID-19 pandemic) and increased support for xenophobic public policies (e.g., surveillance and control of people of different ethnicities during the pandemic). This is in line with the existential threat account outlined by van Prooijen (2019), which posits that sense-making processes resulting from an existential threat only lead to conspiracy beliefs when a person perceives a salient threatening outgroup. Crucially, however, Jolley et al. (2020) showed that the exposure to conspiracy theory promotes prejudice and discrimination not only to the specific group implicated in the conspiracy theory (e.g., Jews), but it also spreads to other social outgroups (such as Asians, Arabs, etc.). As authors noted, this is likely due to the attitude generalization process, in which both positive and negative attitudes toward primary social outgroups (for example, via positive or negative contact with that outgroup) tend to spread to other uninvolved outgroups as well (Shook et al., 2007; Stark et al., 2013).

Moreover, according to the existential threat theory, under some conditions, one’s own government may actually be perceived as the powerful antagonistic outgroup within society which can form the center of conspiracy theorizing when people feel threatened and lacking control (van Prooijen, 2019). Governments in many countries are responsible for communicating the key information about the pandemic, as well as for issuing recommendations and health-preventive regulations to limit the spread of COVID-19. As such, the government may be the most obvious scapegoat for channeling the feelings of anxiety, lack of control, and even anger stemming from the COVID-19 pandemic. Endorsement of conspiracy theories about COVID-19 was previously shown to be associated with lower trust in one’s government, which in turn lead to lower support for and adherence to health preventive measures (Pavela Banai et al., 2020; Pummerer et al., 2021).

Therefore, people who believe COVID-19 conspiracy theories may strongly feel that the health preventive measures are not necessary, because they do not trust their government and health officials, or because some conspiracy theories (e.g., “COVID-19 is a hoax/ordinary flu”) downplay the seriousness of COVID-19 (van Mulukom et al., 2020). This may lead to frustration about strict regulations to prevent the spread of COVID-19, including the closing of non-essential businesses, or curfews, and other long-term constraints to everyday functioning, which may increase the feelings of financial threat, job loss, or social isolation. Such strains resulting from the loss of something important or failure to attain one’s goals are well known to induce anger and sometimes violence (Agnew, 2009).

Conspiracy theories have been linked to negative social outcomes long before COVID-19 pandemic. For example, people who endorse conspiracy theories tend to experience a lack of sociopolitical control and feelings of powerlessness and anomie (Abalakina-Paap et al., 1999; Imhoff and Bruder, 2014). This is why they may believe that they are not capable of influencing sociopolitical events through traditional means, i.e., normative political action such as voting or organized protests. Instead, people who endorse conspiracy theories and/or believe that the world is governed by secret plot tend to prefer non-normative and violent political actions, such as physical attacks, illegal and violent protests, or destruction of property (Imhoff et al., 2021; Levinsson et al., 2021). Recently, in the context of COVID-19 pandemic, Jolley and Paterson (2020) showed that 5G conspiracy beliefs were associated with higher anger, which in turn related to higher justification and willingness to engage in violence – specifically, arson attacks aimed at 5G masts. This effect was especially pronounced among more paranoid individuals, who believed themselves to be personally targeted by the perceived conspiracy motives.

### Present Research

To sum up, in Study 1, which took place early in the pandemic in Slovakia (between April 21st and April 27th 2020, Slovakia had relatively few cases of COVID-19 at that time, the government issued strict preventive regulations which contributed to a mild first wave of the pandemic in Slovakia), we examined conspiracy theories regarding China’s alleged role in creating and spreading the new coronavirus as well as more generic COVID-19 conspiracy theory accounts. We tested the assumption that such conspiracy theories will be related to prejudice toward not only Chinese people, but also other groups associated with the spread of the disease during that time of the pandemic (Italians and Roma people in Slovakia). The data collection for Study 2 took place in November 2020 (between November 2nd and November 6th 2020, the number of infections were raising sharply since the end of summer and eventually this turned into a very severe second wave of the COVID-19 pandemic in Slovakia) when conspiracy theories blaming China or United States for the pandemic were mostly replaced by narratives about the role of the government in spreading COVID-19. We, therefore, surveyed COVID-19 and generic conspiracy belief endorsement to see whether it was associated with decreased prosocial behavior, lower trust in government regulations, higher anger and hostility, and increased justification and willingness to engage in violent actions and not following government health-preventive recommendations. Moreover, in both studies, we also explored the role of exposure to news about COVID-19 and feelings of anxiety and lack of control as potential predictors of COVID-19 conspiracy theory endorsement.

## Study 1

In Study 1, we examined distinct patterns of relationships between generic and China-specific COVID-19 conspiracy beliefs and prejudiced views of three social outgroups mainly associated with COVID-19 pandemic in Slovakia, i.e., Chinese, Italian, and Roma people. The first two groups were selected because of their connection to the COVID-19 pandemic (at the time when Study 1 was conducted, China and Italy were among the countries most severely affected by COVID-19 and received large media coverage in relation to the pandemic), and their inclusion in a previous related study by Oleksy et al. (2021). Roma people were included because they represent perhaps the most marginalized social minority group in Slovakia and at the time of the study, there was a repeating coverage in Slovak media of cases in which COVID-19 spread rapidly among primarily Roma communities, which is why Roma people were considered as the greatest danger in terms of the spread of the virus within their community and outside to majority^1^.

We included generic COVID-19 conspiracy theories (e.g., “COVID-19 is a bioweapon,” “COVID-19 is only a fabrication”) as well as several conspiracy theories related specifically to the alleged role of China in creating and spreading the new coronavirus. As conspiracy theory beliefs are known to promote prejudice that spreads across different social outgroups (Jolley et al., 2020), we wanted to see whether only China-specific conspiracy theories will be associated with prejudice toward Chinese people, or whether there would be more general associations with other social groups (Italian and Roma people) and other, more generic, COVID-19 conspiracy theories as well. Finally, we also examined three potential predictors of COVID-19 conspiracy beliefs – exposure to news about the pandemic, and feelings of anxiety and lack of control – based on the previous findings regarding their role in the endorsement of COVID-19 conspiracy theories and increased prejudice toward social groups associated with the pandemic (Sorokowski et al., 2020; Šrol et al., 2021).

## Materials and Methods

### Participants

The link to the online study was distributed via a participant recruitment agency that was hired to contact a sample representative of the Slovak population in terms of gender and age. In total, we collected the data of 501 participants (241 men, 260 women) who were between 18 and 85 years old (*M* = 45.05, *SD* = 15.92). Most of the participants reported having high school diploma education (73.5%), a smaller part had some college/university education (17.8%), and the rest finished elementary education or high school education without a diploma (8.8%).

The study was part of a larger data collection that was run between 21st and 27th April 2020; participants first answered several demographic questions, items regarding news exposure, anxiety and lack of control, conspiracy theories, and prejudice. There were also several other measures not reported here regarding scientific reasoning, numeracy, and attitudes toward science, which were part of a separate research study.

The *post hoc* sensitivity analysis carried out in G*Power 3.1 software (Faul et al., 2007) suggested that with the current sample size, the study had sufficient (80%) statistical power to detect any correlations with an absolute value of *r* > 0.125 with 5% error probability.

### Materials

The descriptive statistics and internal consistency estimates for all measures reported in Study 1 are presented in Table 1. The data, open materials, and Supplementary Material for this study are publicly available at: https://osf.io/jkab7/.

**TABLE 1:** Descriptive statistics and internal consistency estimates for all materials included in Study 1 and 2.

	M	SD	Range	α
*Study 1*				
Generic COVID-19 conspiracy beliefs	2.49	1.02	1–5	0.89
China-specific COVID-19 conspiracy beliefs	2.08	0.90	1–5	0.84
Negative feelings: China	45.8	21.6	0–100	.
Negative feelings: Italy	35.9	21.8	0–100	.
Negative feelings: Roma	63.2	24.7	0–100	.
Social distance: China	2.36	1.53	1–7	0.85
Social distance: Italy	1.97	1.32	1–7	0.84
Social distance: Roma	3.78	1.77	1–7	0.78
Refusal of help: China	2.89	1.73	1–7	.
Refusal of help: Italy	2.48	1.57	1–7	.
Refusal of help: Roma	3.09	2.01	1–7	.
Anxiety	3.34	1.50	1–7	0.89
Lack of control	3.40	1.43	1–7	0.81
COVID-19 news exposure	4.71	1.23	1–7	0.63
*Study 2*				
COVID-19 conspiracy beliefs	2.32	1.10	1–5	0.93
Generic conspiracy beliefs	2.60	0.96	1–5	0.84
Anger and hostility	2.94	1.39	1–7	0.90
Trust in government regulations	4.59	1.97	1–7	.
Decreased prosocial beh.: COVID-19 pandemic	2.15	0.88	1–5	0.65
Decreased prosocial beh.: general	3.99	0.73	1–5	0.71
5G violence justification	2.43	1.68	1–7	.
5G violence willingness	2.24	1.80	1–7	.
Regulations non-compliance justification	2.64	2.10	1–7	.
Regulations non-compliance willingness	2.16	1.91	1–7	.
Violent protest justification	2.36	1.89	1–7	.
Violent protest willingness	2.14	1.85	1–7	.
COVID-19 news exposure	4.63	1.38	1–7	0.67
Anxiety	3.20	1.45	1–7	0.85
Lack of control	3.39	1.53	1–7	0.84

*The table shows means, standard deviations, observed ranges, and internal consistency estimates (Cronbach’s alpha) for all measures included in Study 1 and 2.*

#### Generic and China-Specific COVID-19 Conspiracy Beliefs

COVID-19 conspiracy beliefs were measured by 12 items (e.g., “*COVID-19 is a biological weapon intended to eliminate the overcrowded human population*”), mostly taken from a previous study (Šrol et al., 2021). We also modified some of the previously used items and added new ones which dealt specifically with the alleged role of China in the spread of the coronavirus (“*The Chinese government already has the cure for COVID-19 (coronavirus) but they are keeping it secret*”). This was done to examine whether the patterns of prejudice associated with the China-specific conspiracy beliefs would differ from those of other more generic COVID-19 conspiracy beliefs. Participants responded to all items on a five-point scale and separate mean scores for the eight generic and four China-specific COVID-19 conspiracy beliefs were calculated. Both subscales showed very good internal consistency (α ≥ 0.84).

For all subsequent analysis, we excluded one of the items from the generic COVID-19 conspiracy beliefs which dealt with the COVID-19 hoax theory. As was shown previously (Imhoff and Lamberty, 2020; Šrol et al., 2021), people who endorse the theory that COVID-19 is a hoax, fabrication, or ordinary flu feel understandably less threatened and anxious about the COVID-19 pandemic. This was also the case in the present study which is why we decided to exclude that one item from all subsequent analyses (for more information on this, see Section A of the Supplementary Material). Therefore, we calculated the average rating on seven remaining items of the generic COVID-19 conspiracy beliefs (*M* = 2.49, *SD* = 1.02, α = 0.89). The resulting score was used in all analyses presented below as a measure of participants’ generic COVID-19 conspiracy theory endorsement.

#### Predictors of COVID-19 Conspiracy Beliefs

##### Anxiety

Participants were asked to rate how often they feel scared, nervous, distressed, and upset because of the ongoing COVID-19 pandemic on a seven-point scale. The ratings for the four items were averaged to gain a single composite anxiety score (α = 0.89).

##### Lack of Control

Participants rated four items taken from Šrol et al. (2021) regarding how they felt about the COVID-19 pandemic. Items asked about the feelings of powerlessness and lacking control, or beliefs that we were no longer able to stop the spread of the disease (e.g., “*I am afraid that the new coronavirus has already spread so much that we will no longer be able to stop it*”). Participants gave ratings on a seven-point scale. The average rating of the four items was used as an indicator of the feeling of lack of control (α = 0.81).

##### Exposure to News About COVID-19

Participants were asked to use a seven-point scale to rate three items regarding how often they encounter or look for news about the COVID-19 pandemic, and how much time they spend discussing it with other people (see Sorokowski et al., 2020). An example item was: “*How often do you encounter information about the coronavirus on the internet, in TV, radio, or in newspapers?*”. The average rating on the three items was used as an indicator of exposure to news about COVID-19 (α = 0.63).

#### Prejudice

We measured prejudiced views of Chinese, Italian, and Roma people in three ways: (1) *Feeling thermometer*: Participants were asked to indicate how cold (negative) or warm (positive) are their feelings toward the three social outgroups on a *feeling thermometer* which ranged from 0 (very cold/negative) to 100 (very warm/positive). (2) *Social distance:* we used Bogardus scale of social distance, a common measure of prejudice (e.g., Sorokowski et al., 2020). Participants used a seven-point scale to indicate how comfortable they would be with a member of the three social outgroups being their coworkers, living in their neighborhood, or becoming part of their family through marriage. Ratings on the three items of social distance scale were averaged into a single composite for every one of the three groups. Measures of prejudice were recoded in a way so that a higher score indicates more negative feelings and higher social distance toward an implicated outgroup (Cronbach’s α for the social distance ≥0.78). (3) *Refusal of help:* we measured the refusal to help the three social outgroups by asking participants to imagine that the Slovak government would want to send material or humanitarian help to Chinese, Italian, or Roma people in need. Participants were asked to rate the degree to which they agreed with providing such help on a seven-point scale. The scores were recoded in a way that higher values reflect stronger refusal to help.

## Results and Discussion

As the first step in our analyses, we scrutinized the correlations between all main variables in Study 1. Then, we focused more specifically on the potential predictors of COVID-19 conspiracy beliefs; we conducted two hierarchical linear regressions where we examined whether news exposure, anxiety, and lack of control predict those beliefs over and above demographic factors (age, gender, and education). Finally, we examined the associations between China-specific and generic COVID-19 conspiracy beliefs and negative feelings, social distance, and refusal of help to Chinese, Italian, and Roma people. Using hierarchical linear regressions and taking into account the demographic factors, we wanted to see whether prejudiced views of the three social outgroups were better predicted by China-specific or rather generic COVID-19 conspiracy beliefs.

Before testing our hypotheses, we also examined the prevalence of conspiracy beliefs in our sample (full descriptions of the endorsement of every COVID-19 conspiracy belief item are presented in Section A in the Supplementary Material). The endorsement of individual conspiracy theories ranged from 5.2 to 32.5%. While conspiracy theories regarding China’s deliberate involvement in production and spread of the virus, or its malevolent concealment of effective treatment were least popular in Slovak participants (only 5.2 to 14.2% of our sample endorsed these suspicions), almost third of the sample (28.1 to 32.5%) endorsed beliefs, such as coronavirus being a bioweapon or artificially created to strengthen people’s dependency on pharmaceutical companies; by far the most prevalent was the belief that the virus could have been stopped early but BigPharma made business out of it. Conspiracy beliefs about the involvement of the United States in the pandemic ranged from 18.4 to 21.4%, suggesting that people in Slovakia are generally more suspicious toward Western democracies, especially United States, than to communist regimes. Interestingly, at the beginning of the pandemic 15.2% believed that COVID-19 is a hoax.

### Correlations Between the Main Variables in Study 1

Firstly, we focused on the associations between COVID-19 conspiracy beliefs and their potential predictors. As can be seen from Table 2, anxiety and lack of control were associated with higher belief in both generic and China-specific conspiracy theories. Although the correlations seemed to be a bit more pronounced in the case of lack of control than anxiety, they all amounted to relatively weak or at best moderate correlations. Concerning the exposure to news about COVID-19, this variable showed no significant association with either of the conspiracy belief indicators, but it was substantially associated with both higher anxiety and lack of control (see also Sorokowski et al., 2020), which makes it potentially interesting as a predictor since both of the latter variables are known to be associated with the endorsement of conspiracy beliefs. Notably, people who reported being more exposed to news about COVID-19 had slightly less negative feelings and were less willing to refuse help to Italian and Roma people.

**TABLE 2:** Pairwise correlations for all variables in Study 1.

	1.	2.	3.	4.	5.	6.	7.	8.	9.	10.	11.	12.	13.
1. Anxiety	1												
2. Lack of control	**0.57**	1											
3. COVID-19 news exposure	**0.43**	**0.33**	1										
4. Generic conspiracy beliefs	**0.12**	**0.24**	−0.06	1									
5. China-specific conspiracy beliefs	**0.20**	**0.28**	0.04	**0.64**	1								
6. China: negative feeling	0.07	0.08	0.00	0.03	**0.11**	1							
7. China: social distance	0.05	**0.09**	0.01	**0.21**	**0.30**	**0.27**	1						
8. China: refusal of help	0.08	**0.11**	−0.07	0.05	**0.18**	**0.28**	**0.20**	1					
9. Italy: negative feeling	−0.06	0.04	**−0.13**	**0.10**	**0.13**	**0.55**	**0.11**	**0.23**	1				
10. Italy: social distance	0.00	0.06	−0.03	**0.17**	**0.24**	**0.17**	**0.74**	**0.12**	**0.22**	1			
11. Italy: refusal of help	0.02	0.05	**−0.16**	**0.16**	**0.17**	**0.13**	**0.12**	**0.66**	**0.28**	**0.18**	1		
12. Roma: negative feeling	0.05	0.04	**−0.09**	**0.20**	**0.11**	**0.33**	**0.10**	**0.10**	**0.29**	0.08	**0.13**	1	
13. Roma: social distance	0.09	0.08	−0.04	**0.20**	**0.19**	0.09	**0.53**	0.09	0.05	**0.40**	0.09	**0.43**	1
14. Roma: refusal of help	−0.02	0.05	**−0.22**	**0.28**	**0.18**	**0.10**	**0.14**	**0.41**	**0.16**	**0.10**	**0.44**	**0.35**	**0.26**

*Correlations are based on 501 observations. Significant correlations (p < 0.05) are presented in bold. Correlations with absolute value of r > 0.088 are significant at p < 0.05, values of r > 0.115 are significant at p < 0.01, and values of r > 0.147 are significant at p < 0.001.*

Next, we examined whether China-specific and generic COVID-19 conspiracy theories are associated with negative feelings, social distance, and refusal of help for Chinese, Italian, and Roma people. As was evident from the strong intercorrelation between the two conspiracy belief variables, people endorsing generic COVID-19 conspiracy theories also endorsed more specific conspiracy theories about China’s alleged involvement in creating and spreading COVID-19. However, China-specific conspiracy theories were less strongly endorsed in comparison with the more generic ones, *t*(500) = 10.2, *p* < 0.001, *d* = 0.46. It was also evident that the patterns of correlations with prejudice variables were somewhat different between generic and China-specific conspiracy theory beliefs. As can be seen from Table 2, negative feelings, social distance, and refusal of help for Chinese people were correlated more strongly with China-specific than generic COVID-19 conspiracy beliefs (please, see Section E in the Supplementary Material for the actual comparison of correlations between these variables). The reverse was true with regard to prejudiced views of Roma people. Finally, both types of conspiracy theory beliefs were correlated to approximately the same extent with negative feelings, social distance and refusal of help for Italian people. However, the correlations between conspiracy theory endorsement and prejudiced views of the three social groups amounted to relatively weak or moderate effect sizes. After all, prejudice is a complex social phenomenon that is likely to be influenced by many other factors besides the current spread of conspiracy beliefs (e.g., Fiske, 1998; Triana et al., 2015; Cowling et al., 2019).

### News Exposure, Anxiety and Lack of Control as Predictors of COVID-19 Conspiracy Beliefs

In order to more specifically disentangle the roles of anxiety, lack of control, and the exposure to news about COVID-19 as predictors of beliefs in conspiracy theories about the pandemic, we have conducted two hierarchical linear regressions, one for generic and the other for China-specific conspiracy beliefs as the outcome variables. In both models, we first entered demographic variables (age, gender, education) at the first step of the regression and then, in the second step, we included anxiety, lack of control, and news exposure to see whether any of these variables predicts conspiracy beliefs after controlling for demographic factors. Even though the news exposure was shown to be uncorrelated with either of the conspiracy belief variables, we included it in the analysis as it was shown to be moderately associated with both anxiety and lack of control and these interrelationships may have precluded the more complex results regarding the connections between these three predictor variables and the belief in COVID-19 conspiracy theories.

As can be seen from Table 3, both generic and China-specific COVID-19 conspiracy beliefs were predicted to the approximately same extent by higher feelings of lack of control even after accounting for demographic factors. Presumably due to strong mutual overlap between the feelings of anxiety and lack of control and consistently with the relatively weak correlations between the two conspiracy belief indicators and anxiety, this latter variable did not show up to predict any additional variance in generic or China-specific COVID-19 conspiracy beliefs at the second step of the regression. However, the belief in generic COVID-19 conspiracy theories was also negatively associated with exposure to news about COVID-19. This is interesting as it suggests that those people that report being more exposed to news about the COVID-19 pandemic perhaps stay more up to date with the official information about the pandemic and thus might endorse fewer conspiracy beliefs about COVID-19. On the other hand, news exposure was moderately associated with higher feelings of anxiety and lack of control (Table 2) which are positively correlated with the endorsement of conspiracy beliefs. Thus, exposure to news about COVID-19 can be associated with both beneficial and negative outcomes (we return to this point below). Although higher education turned up to predict lower belief in generic conspiracy theories and female gender predicted higher belief in China-specific COVID-19 conspiracy beliefs, the proportion of variance explained by these factors was very low (<2%).

**TABLE 3:** The results of hierarchical linear regression predicting China-specific, generic COVID-19, as well as non-COVID-19 generic conspiracy beliefs in Study 1 and 2.

	China-specific COVID-19 conspiracy beliefs (Study 1)		Generic COVID-19 conspiracy beliefs (Study 1)		Generic COVID-19 conspiracy beliefs (Study 2)		Generic (non-COVID-19) conspiracy beliefs (Study 2)	
Predictors	β	95% CI	β	95% CI	β	95% CI	β	95% CI
*Step 1*	Δ*R^2^* = *0.023*		Δ*R^2^* = *0.016*		Δ*R^2^* = *0.085*		Δ*R^2^* = *0.057*	
Age	−0.02	[−0.11, 0.06]	0.08	[−0.00, 0.17]	**0.15**	**[0.09, 0.21]**	**0.11**	**[0.05, 0.17]**
Gender	**0.13**	**[0.04, 0.21]**	0.01	[−0.07, 0.10]	0.06	[−0.01, 0.11]	−0.03	[−0.09, 0.03]
Education	−0.07	[−0.16, 0.01]	**−0.09**	**[−0.18, −0.00]**	**−0.22**	**[−0.28, −0.16]**	**−0.18**	**[−0.24, −0.12]**
*Step 2*	Δ*R^2^* = *0.074*		Δ*R*^2^ = 0.079		Δ*R^2^* = *0.067*		Δ*R^2^* = *0.046*	
COVID-19 news exposure	−0.07	[−0.16, 0.03]	**−0.17**	**[−0.27, −0.08]**	**−0.27**	**[−0.34, −0.21]**	**−0.21**	**[−0.27, −0.15]**
Anxiety	0.07	[−0.04, 0.18]	0.05	[−0.06, 0.16]	**0.09**	**[0.03, 0.15]**	**0.10**	**[0.04, 0.17]**
Lack of control	**0.25**	**[0.14, 0.35]**	**0.27**	**[0.16, 0.37]**	0.03	[−0.03, 0.10]	**0.08**	**[0.01, 0.14]**
*Full model*	*R^2^* = *0.103*		*R^2^* = *0.095*		*R^2^* = *0.153*		*R^2^* = *0.103*	

*The table shows the results of four hierarchical linear regressions predicting China-specific and generic COVID-19 conspiracy beliefs, as well as generic (not related to COVID-19) conspiracy beliefs with several demographic (Step 1) and cognitive and emotional predictors (Step 2). Standardized regression coefficients (β’s) and their 95% confidence intervals are presented for every predictor in the final model. Also, the table shows the change in R^2^ for the both steps of the model as well as the final model. Significant predictors (p < 0.05) are presented in bold. Gender was coded as 1 = “men,” 2 = “women.”*

### COVID-19 Conspiracy Beliefs as Predictors of Prejudiced Views Against the Three Social Outgroups

Finally, we conducted several hierarchical linear regression analyses to help us better understand the patterns of results regarding the generic and China-specific COVID-19 conspiracy theory endorsement as predictors of prejudiced views of Chinese, Italian, and Roma people. For the ease of the analyses, we calculated composite variables representing the prejudiced views of every one of the social groups by averaging z-scores for the three social distance, one refusal of help, and one negative feeling thermometer score for every group separately (each composite variable had acceptable reliability, α’s between 0.70 and 0.75). These prejudice composites were used as outcome variables in three regressions (separate regression for every social outgroup). Two blocks of predictors were used: demographic factors (age, gender, education) were controlled for in the first step of the analysis and then we entered generic and China-specific COVID-19 conspiracy beliefs as two predictors at the second step of the regression.

The results of the regression analyses are presented in Table 4. The findings regarding the roles of generic and China-specific COVID-19 conspiracy beliefs in prejudice complement the correlational results presented above. Generic COVID-19 conspiracy theories only independently predicted prejudiced views of Roma people. On the other hand, China-specific conspiracy beliefs independently predicted prejudice toward Chinese and Italian people. Consistently with the correlation analysis above, the regression coefficients showed weak to moderate effect sizes and explained about 10% of the variance in prejudice against the three social groups even after accounting for the demographic factors. This likely reflects the fact that, as shown in a meta-analysis by Cowling et al. (2019), there are many factors besides conspiracy theory endorsement which may be associated with higher prejudice, such as being male, religious, nationalistic, conservative, less educated, and endorsing right-wing authoritarianism.

**TABLE 4:** The results of hierarchical linear regression predicting prejudice against Chinese, Italian, and Roma people.

	China prejudice		Italy prejudice		Roma prejudice	
Predictor	β	95% CI	β	95% CI	β	95% CI
*Step 1*	Δ*R^2^* = *0.008*		Δ*R^2^* = *0.007*		Δ*R^2^* = *0.006*	
Age	−0.05	[−0.13, 0.04]	−0.07	[−0.16, 0.01]	−0.05	[−0.14, 0.03]
Gender	−0.00	[−0.09, 0.08]	−0.03	[−0.12, 0.06]	−0.02	[−0.11, 0.06]
Education	0.03	[−0.05, 0.12]	−0.01	[−0.09, −0.08]	−0.03	[−0.12, −0.06]
*Step 2*	Δ*R^2^* = *0.107*		Δ*R*^2^ = 0.076		Δ*R^2^* = *0.079*	
Generic COVID-19 conspiracy beliefs	−0.03	[−0.14, 0.08]	0.07	[−0.05, 0.18]	**0.24**	**[0.12, 0.35]**
China-specific COVID-19 conspiracy beliefs	**0.35**	**[0.24, 0.46]**	**0.23**	**[0.12, 0.34]**	0.07	[−0.04, 0.18]
*Full model*	*R^2^* = *0.115*		*R^2^* = *0.082*		*R^2^* = *0.084*	

*The table shows the results of three hierarchical linear regressions predicting prejudice against Chinese, Italian, and Roma people with several demographic (Step 1) and two conspiracy belief predictors (Step 2). Standardized regression coefficients (β’s) and their 95% confidence intervals are presented for every predictor in the final model. Also, the table shows the change in R^2^ for the both steps of the model as well as the final model. Significant predictors (p < 0.05) are presented in bold. Gender was coded as 1 = “men,” 2 = “women.”*

Based on the present results it seems that Slovak people do not have a strong prejudice against Chinese or Italian people, at least when compared to Roma people (negative feelings, social distance, and refusal of help were all significantly higher toward Roma people than Chinese and Italian people; see Section B of the Supplementary Material). However, those that endorsed the more specific, and less common, conspiracy theories about the alleged role of China held also more prejudiced views of Chinese and Italian people – two foreign groups most associated with the COVID-19 pandemic at the time. For example, as was found by Sorokowski et al. (2020), people who felt more anxious about the COVID-19 pandemic had more negative attitudes about Italians. Adopting conspiracy beliefs may be one of the mechanisms which explain why higher anxiety from the pandemic is associated with stronger prejudice toward groups associated with the pandemic. On the other hand, more generic COVID-19 conspiracy theories do not predict prejudice of Chinese and Italian people, who are, in contrast with Roma people, not traditionally viewed as a threat by the Slovak majority population. The more commonly endorsed generic COVID-19 conspiracy theories were more strongly associated with prejudice toward Roma people likely because they represent the group that is traditionally used as a scapegoat (Silverman, 1995; Batueva, 2012; Kapralski, 2016) against which the frustrations and anxieties of Slovak majority people can be channeled. These differences in patterns of results between the two types of conspiracy theories are notable mainly if we take into account the strong correlation between the China-specific and generic COVID-19 conspiracy beliefs.

## Study 2

While in the first study, we focused on prejudiced attitudes against groups most associated with the coronavirus at the time, as the most obvious instance of negative social outcomes accompanying conspiracy theories at the time, as the pandemic progressed the mood in society shifted. While the beginning of pandemic was marked with high solidarity and cohesion and the targets of the conspiracy theories were external, dissatisfaction with government’s management of the pandemic lead to activation of citizens and political opposition and proliferation of conspiracy theories that targeted proposed measures to contain pandemic. Therefore, we also changed the focus of the negative social outcomes that we examined in the second study. Specifically, we included justification of and willingness to engage in violent attacks on 5G masts, violent anti-government protests, and non-compliance with government regulations. State anger and hostility was included as a further predictor of the justification and willingness to engage in violent attacks, protests, and regulations non-compliance (Jolley and Paterson, 2020). Lower trust in government regulations, on the other hand, was further hypothesized to predict the non-compliance with health preventive measures (Pavela Banai et al., 2020; Pummerer et al., 2021), as well as violent protests against these government regulations. We also explored decreased prosocial behavior as another potential dimension of negative social outcomes associated with COVID-19 conspiracy theories, based on some previous partial evidence for the link between conspiracy theory endorsement and prosociality (van der Linden, 2015; Oleksy et al., 2021). As in Study 1, we also included news exposure and feelings of anxiety and lack of control as potential predictors of COVID-19 conspiracy beliefs.

## Materials and Methods

### Participants

The participants were recruited through an agency that send out the link to the online study to a sample that was representative of the Slovak population in terms of gender, age, and education. In total, we collected the data of 1024 participants (486 men, 536 women, 2 preferred not to disclose their gender) who were between 18 and 81 years old (*M* = 44.2, *SD* = 15.3). Concerning education, most of the participants reported having finished high school with a diploma (39.1%), the rest either had some college degree (22.9%) or finished either elementary education (6.3%) or high school without a diploma (31.3%).

The study was part of a larger data collection that was run between 2nd and 6th November 2020. Participants first answered several demographic questions including experience with COVID-19, items regarding news exposure, anxiety, lack of control, conspiracy theories, decreased prosocial behavior, violence acceptance, and trust in government regulations. They also answered several other measures not reported here regarding well-being, scientific reasoning, numeracy, and attitudes toward science, vaccination attitudes and vaccination intentions, personality and following the government anti-pandemic regulations, which were part of two separate research projects (Čavojová et al., 2021; Kohút et al., 2021).

The *post hoc* sensitivity analysis carried out in G*Power 3.1 software (Faul et al., 2007) suggested that with the current sample size, the study had sufficient (80%) statistical power to detect any correlations with an absolute value of *r* > 0.088 with 5% error probability.

### Materials

The descriptive statistics and internal consistency estimates for all measures reported in Study 2 are presented in Table 1. The data, open materials, and Supplementary Material for this study are publicly available at: https://osf.io/jkab7/.

#### Generic and COVID-19 Conspiracy Beliefs

##### COVID-19 Conspiracy Beliefs

Conspiracy beliefs about COVID-19 were measured by ten items, which participants rated on a five-point scale. Because the nature of popular conspiracy beliefs pertaining to COVID-19 changed from when our Study 1 was conducted, most of the items here were newly created. For example, in the early months of the pandemic, the most salient conspiracy beliefs concerned the origin of the virus (China/United States), while in the time of Study 2, they were mostly replaced by conspiracy theories regarding the government’s malevolent intentions behind testing and vaccination (e.g., “*Governments want to use compulsory vaccination against COVID-19 to achieve monitoring of the population”).* The average score was used as an indicator of COVID-19 conspiracy beliefs (α = 0.94). As in Study 1, we excluded one item (COVID-19 hoax theory) from the composite COVID-19 conspiracy theory score (see Section A of the Supplementary Material), and only analyzed the remaining nine items here (*M* = 2.32, *SD* = 1.10, α = 0.93), as well as in the regression analyses below.

##### Generic Conspiracy Beliefs

Generic conspiracy beliefs (not pertaining to COVID-19) were measured using six items taken from the questionnaire by Šrol (2021). These items dealt with well-known conspiracy motives such as perpetrators of the events on 9/11, or chemtrails (e.g., “*Exhaust seen in the sky behind airplanes is actually chemicals sprayed by the government for sinister reasons*”). Participants rated the items on a five-point scale and the average score was used as an indicator of generic conspiracy beliefs (α = 0.84).

#### Predictors of COVID-19 Conspiracy Beliefs

The materials used to measure news exposure, anxiety, and lack of control were almost identical to the ones used in Study 1.

#### Additional Predictors of Negative Social Outcomes

##### Anger and Hostility

Participants were asked to rate to what extent (on a scale from 1 “very slightly” to 5 “very strongly”) they lately feel the following emotions: upset, hostile, irritable, angry, furious, and annoyed. The average rating for the six items was used as a composite anger and hostility score (α = 0.90).

##### Trust in Government Health-Preventive Regulations

We asked participants to indicate whether they think that the government health-preventive regulations related to the spread of COVID-19 are well-grounded or not on a seven-point scale.

#### Negative Social Outcomes Associated With COVID-19 Pandemic

##### Decreased Prosocial Behavior

We used four items to measuring decreased prosocial behavior specifically during the COVID-19 pandemic (e.g., a reverse-scored item: “*I donate to charity for people affected by pandemic*.”) and five items measuring lower prosocial behavior in general [e.g., “*When I see someone on the street needing help (e.g., looking lost), I try to avoid them.*”]. Participants indicated their agreement on a five-point scale. We calculated the average score for decreased COVID-19 specific prosocial behavior and general prosocial behavior (α ≥ 0.65).

##### Justification and Willingness to Engage in Violent Attacks on 5G Masts, Violent Anti-government Protests, and Non-compliance With Government Health Regulations

We presented participants with news stories which described three recent and real events: violent protest against Slovak government regulations pertaining to COVID-19, arsons at 5G mobile towers in Great Britain, and a reported case from a Slovak hospital when a pregnant patient refused to follow the official safety guidelines (taking a test for COVID-19, wearing a face-mask), thereby obstructing her admittance to a maternity ward. The full wording of these scenarios is presented in Section C of the Supplementary Material. After each scenario, participants were asked whether the behavior described in the news stories was justified in their view (scale between 1 = “*completely unjustified*” and 7 “*completely justified*”) and whether they would consider engaging in such behavior themselves (scale between 1 = “*definitely not*” and 7 “*definitely yes*”).

## Results and Discussion

As the first step in our analyses, we examined the correlations of cognitive and emotional predictors (news exposure, anxiety, and lack of control), COVID-19 and generic conspiracy beliefs, decreased prosocial behavior, trust in government health-preventive regulations, anger and hostility, and justification and willingness to take part in violent actions and acts of non-compliance with government regulations. Then, as in Study 1, we examined the predictors of COVID-19 conspiracy theories – news exposure, feelings of anxiety and lack of control – via a hierarchical linear regression analysis while controlling for demographic factors. Finally, we conducted three regression analyses to examine demographic variables, anger and hostility, trust in government regulations, and COVID-19 conspiracy beliefs as predictors of justification and willingness to take part in 5G violence, violent anti-government protest, and regulations non-compliance.

Before testing our hypotheses, we also examined the prevalence of conspiracy beliefs in our sample (full descriptions of the endorsement of every COVID-19 conspiracy belief item are presented in Section A in the Supplementary Material). The endorsement of individual conspiracy theories ranged from 11.5 to 34.7%. Again, as in Study 1 the most prevalent was the belief that the virus could have been stopped early but BigPharma made business out of it (34.7%) and that coronavirus was developed as a bioweapon (32.6%). 29.6% believed that pandemic was created artificially to enable governments better control of their citizens. Belief that COVID-19 is a hoax increased and was endorsed by 20.1% of our sample. The more prevalent than hoax theory was belief that vaccination is a mean to monitor citizens (22.4%). The belief that government conceals the true numbers of death to prevent panic decreased to 17.4%, probably due to radically worse situation in November than in April 2020. Other newly included conspiracy theories were endorsed by less than 20% of people: these included 5G conspiracy theory (18.7%), genocide of domestic citizens to make space for immigrants (15.6%), Bill Gates using vaccines to insert microchips (13.3%), national antigen testing as a way to gain DNA of citizens (11.5%).

### Correlations Between the Main Variables in Study 2

Firstly, we inspected the correlations between news exposure, anxiety, lack of control, and the endorsement of conspiracy theories. As can be seen from Table 5, the patterns of correlations with COVID-19 predictors are a bit different from those observed in Study 1. Specifically, while the news exposure is associated with stronger feelings of anxiety and lack of control, the correlations between those feelings and COVID-19 conspiracy beliefs are non-significant. The only significant, albeit very weak, correlation was found between anxiety and generic conspiracy beliefs. On the other hand, exposure to news about COVID-19 turned out to be associated with lower belief in both generic and COVID-19 conspiracy theories.

**TABLE 5:** Pairwise correlations for all variables in Study 2.

	1.	2.	3.	4.	5.	6.	7.	8.	9.	10.	11.	12.	13.	14.
1. Anxiety	1													
2. Lack of control	**0.35**	1												
3. COVID-19 news exposure	**0.25**	**0.27**	1											
4. COVID-19 conspiracy beliefs	0.04	0.01	**−0.25**	1										
5. Generic conspiracy beliefs	**0.07**	0.05	**−0.18**	**0.75**	1									
6. Anger and hostility	**0.79**	**0.24**	**0.13**	**0.13**	**0.15**	1								
7. Trust in gov. regulations	0.02	**0.18**	**0.32**	**−0.59**	**−0.41**	**−0.15**	1							
8. 5G violence justification	**0.08**	−0.01	**−0.15**	**0.56**	**0.50**	**0.14**	**−0.31**	1						
9. 5G violence willingness	0.04	0.04	**−0.10**	**0.48**	**0.42**	**0.08**	**−0.25**	**0.67**	1					
10. Violent protest justification	**0.08**	**−0.07**	**−0.20**	**0.51**	**0.40**	**0.18**	**−0.48**	**0.50**	**0.46**	1				
11. Violent protest willingness	0.06	**−0.11**	**−0.19**	**0.53**	**0.42**	**0.18**	**−0.52**	**0.45**	**0.49**	**0.74**	1			
12. Non-compliance justification	−0.01	**−0.21**	**−0.23**	**0.60**	**0.48**	**0.11**	**−0.56**	**0.44**	**0.40**	**0.54**	**0.56**	1		
13. Non-compliance willingness	−0.04	**−0.12**	**−0.29**	**0.41**	**0.27**	0.04	**−0.44**	**0.31**	**0.28**	**0.36**	**0.42**	**0.46**	1	
14. Decreased prosocial behavior (pandemic)	0.06	**0.14**	**0.16**	−0.02	−0.02	−0.01	**0.10**	0.05	0.03	0.05	−0.02	−0.03	0.06	1
15. Decreased prosocial behavior (general)	−0.02	−0.03	**0.13**	−0.01	−0.02	**−0.14**	**0.06**	**−0.07**	**−0.07**	**0.12**	**−0.07**	−0.01	**0.08**	**0.22**

*Correlations are based on 1024 observations. Significant correlations (p < 0.05) are presented in bold. Correlations with absolute value of r > 0.062 are significant at p < 0.05, values of r > 0.081 are significant at p < 0.01, and values of r > 0.103 are significant at p < 0.001.*

Next, we move on to the correlations between conspiracy theory endorsement, anger and hostility, trust in government regulations, and negative social outcomes. The patterns of correlations with various negative social outcome variables are almost indistinguishable between COVID-19 and generic conspiracy beliefs (somewhat weaker correlations for the generic conspiracy beliefs may be due to lower reliability of its measurement in comparison with COVID-19 conspiracy beliefs). This is consistent with the very high correlation between the two types of conspiracy beliefs measures. Indeed, people who endorse some conspiracy theories are usually found to endorse also other, unrelated conspiracy beliefs (e.g., Swami et al., 2011; Miller, 2020).

Both conspiracy belief variables were, consistently with our expectations, correlated with slightly higher feelings of anger and hostility, substantially lower trust in government regulations, and also considerably higher tendency to justify violence and non-compliance with COVID-19 regulations and willingness to take part on such actions (Table 5). However, we did not find any relationship between generic or COVID-19 conspiracy beliefs and reported prosocial behavior.

### News Exposure, Anxiety and Lack of Control as Predictors of COVID-19 Conspiracy Beliefs

Using the same regression model as in Study 1, we examined whether anxiety, lack of control, and exposure to news about COVID-19 predicts the endorsement of COVID-19 and generic conspiracy beliefs over and above age, gender, and education. As can be seen from the right columns of Table 3, the results were a bit different from those observed a couple of months earlier in Study 1. That is, news exposure was moderately negatively associated with COVID-19 conspiracy beliefs. While lack of control – which turned up to be the strongest predictor in the same analysis in Study 1 – was not significantly associated with higher conspiracy beliefs, anxiety weakly positively predicted higher endorsement of COVID-19 conspiracy theories. In comparison with Study 1, the predictive power of news exposure seemed to grow relatively stronger at the expense of emotional factors (anxiety and lack of control). On the other hand, generic conspiracy beliefs were significantly predicted both by lower exposure to COVID-19 news and higher anxiety and lack of control. Also importantly, both higher age and lower education predicted higher belief in COVID-19 and generic conspiracy beliefs.

### COVID-19 Conspiracy Beliefs, Anger and Hostility, and Trust in Regulations as Predictors of Negative Social Outcomes

Finally, we conducted three hierarchical linear regression analyses to examine the predictors of justification of and willingness to take a part in violence against 5G masts, violent anti-government protests, and acts of non-compliance with COVID-19 regulations. For the ease of analyses, we averaged the rating for justification and willingness for every type of behavior separately to create three negative social outcomes. All regressions were constructed in two steps. At the first step, we entered age, gender, and education and then, we included COVID-19 conspiracy beliefs, anger and hostility, and trust in regulations to see whether they predict any variance in these negative social outcomes over and above the demographic factors. Although we have used COVID-19 conspiracy theory endorsement in all three models as a key predictor, note that all regressions were also rerun with generic conspiracy theory belief variable and the results were similar to those presented below. The three regression models with generic conspiracy beliefs as predictors can be found in Section D of the Supplementary Material. As decreased prosocial behavior was not significantly correlated with either of our conspiracy belief variables, we did not construct regression models for the two prosocial behavior variables as outcomes.

The results of the three regression models are shown in Table 6. Building on the recent study by Jolley and Paterson (2020), we analyzed whether COVID-19 conspiracy beliefs along with higher anger and hostility predict increased justification of and willingness to take part in violent protests at 5G network masts. Our results indeed show that people who endorse more conspiracy beliefs about COVID-19 perceive violent protest against 5G networks as justified and report being much more willing to take part in such protests. Although COVID-19 conspiracy beliefs were shown above to be associated with somewhat increased anger and hostility, these feelings were not directly related to justification and willingness to engage in 5G violence once the endorsement of COVID-19 conspiracy beliefs was taken into account. Notably, only one of the surveyed conspiracy theory items was actually related to the alleged link between the 5G network and COVID-19. This suggests that conspiracy theories may not only serve to justify violence toward the alleged actors and implicated groups but also increase the general sympathy for more violent, instead of normative sociopolitical actions (e.g., Imhoff et al., 2021; Levinsson et al., 2021). Also, there was a very slight association between lower education level and higher justification and willingness to take part in violent protests against 5G masts. Although the predictive power of education was very weak, perhaps this shows that people with higher education are less likely to believe the link between the spread of COVID-19 and the 5G network and are thus less likely to support protests against building of new 5G masts.

**TABLE 6:** The results of hierarchical linear regression predicting negative social outcomes.

	5G violence justification and willingness		Regulations non-compliance justification and willingness		Violent protest justification and willingness	
Predictor	β	95% CI	β	95% CI	β	95% CI
*Step 1*	Δ*R^2^* = *0.051*		Δ*R^2^* = *0.039*		Δ*R^2^* = *0.045*	
Age	0.03	[−0.02, 0.08]	**−0.05**	**[−0.10, −0.00]**	0.01	[−0.04, 0.06]
Gender	0.04	[−0.01, 0.09]	**−0.06**	**[−0.11, −0.02]**	**−0.08**	**[−0.12, −0.03]**
Education	**−0.05**	**[−0.11, −0.00]**	−0.03	[−0.08, 0.01]	−0.05	[−0.10, 0.00]
*Step 2*	Δ*R^2^* = *0.276*		Δ*R*^2^ = 0.407		Δ*R^2^* = *0.350*	
COVID-19 conspiracy beliefs	**0.54**	**[0.49, 0.59]**	**0.39**	**[0.33, 0.44]**	**0.36**	**[0.29, 0.42]**
Anger and hostility	0.05	[−0.00, 0.10]	−0.02	[−0.07, 0.03]	**0.10**	**[0.06, 0.15]**
Trust in regulations	−	−	**−0.36**	**[−0.41, −0.30]**	**−0.30**	**[−0.36, −0.24]**
*Full model*	*R^2^* = *0.328*		*R^2^* = *0.446*		*R^2^* = *0.394*	

*The table shows the results of three hierarchical linear regressions predicting negative social outcomes with several demographic predictors (Step 1) and variables related to the endorsement of conspiracy beliefs (Step 2). Standardized regression coefficients (β’s) and their 95% confidence intervals are presented for every predictor in the final model. Also, the table shows the change in R^2^ for the both steps of the model as well as the final model. Significant predictors (p < 0.05) are presented in bold. Gender was coded as 1 = “men,” 2 = “women.”*

Concerning the justification of and willingness to engage in acts of non-compliance with government regulations (refusing wearing a facemask when visiting a hospital), the results show that both higher belief in COVID-19 conspiracy theories and lower trust in government regulations were related to this negative social outcome to approximately same extent. This is completely in line with two recent studies which showed that COVID-19 conspiracy beliefs predict reduced support for government regulations and lower compliance with health-protective guidelines both directly and indirectly through lowering trust in one’s government (Pavela Banai et al., 2020; Pummerer et al., 2021). While anger and hostility did not show up as a significant independent predictor in this analysis, both female gender and lower age were associated with lower justification and willingness to take part in non-compliance with preventive regulations. However, it should be stressed that the predictive power of the two demographic factors was negligible in comparison with that of COVID-19 conspiracy beliefs and trust in government regulations (4 vs. 41% explained variance).

In the third regression, we predicted the justification of and willingness to engage in violent anti-government regulation protests. As can be seen from Table 6, the results were similar to the previous regression model. Again, both higher belief in COVID-19 conspiracy theories and lower trust in government regulations both moderately predicted this negative social outcome variable. In this case, however, increased anger and hostility was also predictive of higher justification and willingness to take part in violent anti-government protests. This shows that some COVID-19 conspiracy theory believers may indeed see their government as an antagonistic powerful outgroup (van Prooijen, 2019) to which they direct their frustrations from the pandemic. Indeed, many of the prolific conspiracy theories in Slovakia were directed at the government and the issued regulations. For example, around 20% of participants in this sample believed that the COVID-19 is only a fabrication which the government is intentionally using to restrict people’s civil rights, or that the government wants to use vaccination to achieve the monitoring of its population (see Section A of the Supplementary Material). Among people who endorse similar conspiracy beliefs, trust in health-preventive regulations is probably very low and they may fear the regulations more than the virus itself. Moreover, prolonged frustration from the strict health-preventive measures may lead to anger (Agnew, 2009), which may serve to justify the violent nature of anti-government protests instead of more peaceful forms of sociopolitical action. This would explain the strong link between COVID-19 conspiracy theories, higher hostility, lower trust in government regulations, and justification of and willingness to engage in violent forms of political protest against government regulations.

## General Discussion

During the ongoing COVID-19 pandemic, people face many dangers, from the most obvious health threats, to the economic perils of the slowed-down economy or job loss, reduced social contacts, or even temporary restrictions on their civil rights. It is no wonder that as a response to these threats people experience feelings of anxiety and powerlessness, both of which lead to sense-making processes that can result in various conspiracy theory beliefs (van Prooijen and Douglas, 2017; van Prooijen and van Vugt, 2018; Douglas et al., 2019).

In the two present studies, we examined both cognitive (news exposure) and emotional predictors (anxiety, lack of control) of COVID-19 conspiracy beliefs. Interestingly, we seemed to observe that the exposure to news about COVID-19 had two distinct effects on the endorsement of COVID-19 conspiracy theories. Being exposed to more news about COVID-19 was consistently associated with having fewer conspiracy beliefs both about the new coronavirus and the more generic conspiracy theories not related to the COVID-19 pandemic. This could be because higher exposure to official news could counteract the effect of conspiracy theories and fake news often disguised as “unofficial information.” Indeed, while trying to counteract the effects of various misinformation after people were already exposed to them is notoriously hard, presenting people with reliable information that can counteract the effect of subsequently encountered misinformation is believed to be an effective strategy for dealing with various, including COVID-19 related, misinformation (van der Linden et al., 2020). However, higher news exposure was in both studies also moderately correlated with the extent to which participants felt anxiety and lack of control. This is hardly surprising, as the news about pandemic often include such dire information as the new numbers of infected people and casualties, along with prolonging and/or stepping up the preventive measures against the spread of the new coronavirus. Being constantly exposed to such news might make people feel uneasy and even feel like the COVID-19 pandemic spread to such proportions that it cannot be stopped anymore. Such feelings of anxiety and lack of control were in turn associated with higher endorsement of both generic and China-specific COVID-19 conspiracy theories as well as conspiracy accounts not related to COVID-19 pandemic. However, it should be said that the associations between these two affective variables and conspiracy beliefs were somewhat inconsistent across our two studies. While mostly anxiety predicted COVID-19 and generic conspiracy theory beliefs in Study 2, lack of control was the sole affective predictor of the two conspiracy belief variables in Study 1. It is possible that anxiety and lack of control reflect slightly different emotional response as it unfolded during pandemic: while at the beginning people lack of control could represent acute emotional reaction to the novel situation, as people developed various mechanisms to handle it, the more chronic feeling of anxiety prevailed. Be that as it may, our results seem to be in line with the previous findings that show that feelings of anxiety and/or lack of control are associated with higher endorsement of COVID-19 conspiracy beliefs both in the COVID-19 pandemic context (Biddlestone et al., 2020; Sallam et al., 2020; Šrol et al., 2021), and outside of it (e.g., Grzesiak-Feldman, 2013; van Prooijen and Acker, 2015; Swami et al., 2016).

Our analysis of predictors of COVID-19 conspiracy theories – news exposure, anxiety and lack of control – brings about an important policy implication. While it is notoriously hard to counteract conspiracy theories and other misinformation when people already believe them, proactive communication of official information may serve to inoculate the public and counteract the effects of subsequently encountered misinformation (van der Linden et al., 2020). However, it is imperative that the communication of official information by the government, health officials, and media is done in such a way as not to increase people’s feeling of anxiety and lack of control, which were shown to contribute to a higher endorsement of COVID-19 conspiracy theories (for a review, see van Mulukom et al., 2020). For example, it is possible that the communication style of Slovak prime minister, often criticized for being unpredictable, unclear, or downright dangerous (e.g., Slovak Spectator, 2021a, February 8; 2021b, March 3), may have actually helped the spread of various misinformation related to COVID-19 pandemic rather than contributing to counteracting them.

Moreover, under some conditions, the frustration resulting from the pandemic and its perceived mismanagement by the government may lead to anger and subsequently to acts of aggression and violence (Agnew, 2009). In the two present studies, we showed distinct negative social outcomes associated with the endorsement of COVID-19 conspiracy theories. Study 1 was focused on the potential outcomes of attributing threats of COVID-19 pandemic to social outgroups, to wit, those that themselves struggled the most with the new coronavirus – Chinese, Italian, and Roma people in Slovakia. While prejudice against Chinese and Italian people were relatively rare in Slovakia, it was moderately associated with endorsing conspiracy theories about China’s alleged role in the creation and spreading of COVID-19. Prejudice against Roma people was much more common in the Slovak majority population and was associated with generic COVID-19 conspiracy theories. These findings support the account that conspiracy beliefs may serve the social motive of protecting one’s ingroup against the ostensibly threatening outgroup (Douglas et al., 2017; van Prooijen, 2019), as well as the finding that the prejudices brought about by specific conspiracy theories against particular groups may spread to other outgroups (Jolley et al., 2020), and may be associated even with seemingly unrelated conspiracy theory accounts.

Study 2 examined the justification and willingness to engage in acts of violence, protests, and non-compliance with government regulations related to COVID-19 conspiracy theories at the time when these accounts in Slovakia became much more focused on the alleged role of the Slovak government in spreading the COVID-19. As outlined by van Prooijen (2019), it is not uncommon for people to perceive their own government as a powerful antagonistic outgroup when they feel threatened and lacking control. Consistently with this, we found that COVID-19 and generic conspiracy theories were associated with substantially lowered trust in government regulations, slightly higher anger and hostility, and subsequently with the justification of and willingness to engage in non-compliance with health-preventive regulations and even violent protests and attacks.

However, the relationship between conspiracy beliefs and anger and hostility was relatively modest (similarly as in Jolley and Paterson, 2020), presumably because these feelings were measured by asking participants how they felt lately in general, not specifically in relation to some aspect of COVID-19 pandemic. Also, as was recently shown by Jolley and Paterson (2020), both generic conspiracy beliefs and specific COVID-19 conspiracy beliefs are associated with stronger justification and willingness for violent protests against 5G network masts.

Correlations between both conspiracy belief variables and lower trust in government health-preventive regulations, justification of, and willingness to take part in 5G violence, anti-government violent protests, and acts of non-compliance with government regulations were, in contrast, relatively strong. It is well documented that the endorsement of conspiracy theories about COVID-19 is generally related to lower trust in authorities, such as government and health institutions (Freeman et al., 2020; Pummerer et al., 2021; Šrol et al., 2021), as well as lower adherence to many of the government-issued health-preventive behaviors (Freeman et al., 2020; Pavela Banai et al., 2020; for review, see van Mulukom et al., 2020). Our results complement these findings and extend them also to the domain of more general actions of non-compliance with government regulations and violent protests. Although, not all studies found link between endorsement of conspiracy theories and lowered adherence to health protective behaviors (Imhoff and Lamberty, 2020; Juanchich et al., 2021), it largely depends on the what kinds of behaviors were examined. For example, people believing in conspiracy theories in Juanchich et al. (2021) washed their hands as often as non-believers, but they were still less willing to install the contact-tracing app or get vaccinated, i.e., socially oriented behaviors. Similarly, in Imhoff and Lamberty (2020) belief that coronavirus is bioweapon did not correlate with containment-related behavior, but it correlated self-centered prepping behavior. These findings suggest conspiracy theories have distinct effects.

Considering decreased prosocial behavior, however, we found no relationship between conspiracy beliefs and self-reported behavior either in general or specifically during the COVID-19 pandemic. This is in contrast with the findings of van der Linden (2015), who showed that the exposure to conspiracy theory video about global warming slightly reduced people’s prosocial intentions – donating to charity and volunteering. We asked about more mundane and everyday prosocial behaviors, such as refusing to help others who are in need or not supporting people and businesses that require help during the COVID-19 pandemic. Although the correlations were in the expected direction, they were of negligible size. Similar findings were brought by Oleksy et al. (2021), who found that general COVID-19 conspiracy beliefs did not correlate with pro-sociality, and while the endorsement of government-related COVID-19 conspiracy theories did, its correlation in this regard was very low. Therefore, if any relationship between conspiracy beliefs and lower pro-social behaviors exists, it seems to be weak at best. While the present studies helped us to understand the proliferation of various COVID-19 conspiracy theories and associated negative social outcomes at two different stages of the pandemic, the cross-section design of our studies prevents us from drawing conclusions about the causal effects of COVID-19 conspiracy beliefs. That is, any outcome social variables examined here could be associated with conspiracy theory endorsement in a more intricate way through other confounding variables, or they may help to explain the endorsement of conspiracy beliefs instead of being their consequences. While we cannot eliminate this possibility, we would like to note that our findings complement some previous experimental and longitudinal evidence. For example, an experimental study by Jolley et al. (2020) showed that exposure to conspiracy theories leads to increased prejudice which spreads across social outgroups. On the other hand, longitudinal research of Pummerer et al. (2021) showed that COVID-19 conspiracy theories predicted lower trust in and support of government regulations rather than vice versa. Nevertheless, future experimental and longitudinal research could help to shed further light on the intricate relationships between COVID-19 conspiracy theories and their negative social outcomes.

## Conclusion

While conspiracy theory endorsement is known to be associated with adverse social phenomena (e.g., Jolley and Douglas, 2014; Jolley et al., 2019, 2020), the research on prejudice, support for violence and non-normative political action is so far largely lacking from the studies focusing on COVID-19 conspiracy theories (van Mulukom et al., 2020). As shown here, people who endorse various conspiracy theories about COVID-19 have both higher prejudice toward social groups associated with the pandemic and feel more anger and hostility, lower trust in government regulations, and they report higher intentions to participate in violent actions and non-compliance with preventive regulations. Together, these findings may help to shed light on recent highly publicized violent anti-government and anti-regulation protests which took place all around the Western hemisphere (e.g., Bradley et al., 2020, August 29; Jolley and Paterson, 2020). While there is certainly no easy way to deal with these social consequences, one partial solution can be seen in the communication of official information in a way that also takes into account people’s feelings of frustration, anxiety, and lack of control, that may be channeled into wide-spread conspiracy theory endorsement (van Prooijen, 2019).

## Data Availability Statement

The datasets presented in this study can be found in online repositories. The names of the repository/repositories and accession number(s) can be found below: https://osf.io/jkab7/.

## Ethics Statement

Ethical review and approval was not required for the study on human participants in accordance with the local legislation and institutional requirements. The patients/participants provided their written informed consent to participate in this study.

## Author Contributions

JŠ, VČ, and EB conceived of the presented idea. VČ and JŠ developed the theory. JŠ performed the computations. All authors discussed the results and contributed to the final manuscript.

## Conflict of Interest

The authors declare that the research was conducted in the absence of any commercial or financial relationships that could be construed as a potential conflict of interest.

## Publisher’s Note

All claims expressed in this article are solely those of the authors and do not necessarily represent those of their affiliated organizations, or those of the publisher, the editors and the reviewers. Any product that may be evaluated in this article, or claim that may be made by its manufacturer, is not guaranteed or endorsed by the publisher.

## Footnotes

1 In Slovakia, Roma people are typically ostracized because they differ from the majority by their appearance and segregated living conditions and related poverty. Many of Roma people work abroad (especially in the United Kingdom) and after closing borders in the whole Europe in the spring of 2020 they were returning home in large numbers and they were perceived as a threat to the health of the majority. This sentiment was reflected also in the language used by many political representatives including the Slovak prime minister (e.g., EURACTIV.sk, 2020, July 30; Slovak Spectator, 2020, April 8).
