# Supplementary material for: Finding Someone to Blame: The Link Between COVID-19 Conspiracy Beliefs, Prejudice, Support for Violence, and Other Negative Social Outcomes
Source: Front Psychol. 2022 Jan 14;12:726076. doi: 10.3389/fpsyg.2021.726076 (PMC8795973; doi:10.3389/fpsyg.2021.726076)
Supplement: Supplementary file 1 [file Data_Sheet_1.DOCX]

# SUPPLEMENTARY MATERIAL

**Section A**

**Item-level analysis of predictors of COVID-19 conspiracy beliefs: associations with information exposure and feelings of anxiety and lack of control in Study 1 and Study 2**

As was shown previously, not all COVID-19 conspiracy theories are associated with higher perceived anxiety, or lack of control. One of the popular conspiracy theories about COVID-19 is that it is a hoax, fabrication, or that it is no more dangerous than ordinary flu (i.e. COVID-19 hoax theory; van Mulukom et al., n.d.). People who believe this account understandably feel less threatened, anxious, and/or lacking control with regard to the COVID-19 pandemic (e.g. Imhoff & Lamberty, 2020; Šrol et al., 2021). This is why, prior to the analyses of potential predictors of COVID-19 conspiracy theories – news exposure, anxiety, and lack of control – presented in the main manuscript, we have scrutinized the item-level associations between these predictors and particular conspiracy theory belief items included in Study 1 and Study 2. The results of these analyses are presented in Table S1 and S2.

As can be seen from Table S1, almost all of the generic and China-specific COVID-19 conspiracy theory items in Study 1 were positively correlated with feelings of anxiety and lack of control. As could be expected based on previous results of Šrol et al., (2021), however, the item measuring COVID-19 hoax theory (i.e. “*COVID-19 (coronavirus) is only a fabrication…*”) actually showed an opposite pattern of associations with anxiety and lack of control. This is understandable as people who believe this theory are unlikely to feel threatened by COVID-19 or anxious because of it. Although the patterns of correlations between predictors and particular COVID-19 conspiracy theories were much less pronounced in Study 2 (see Table S2), COVID-19 hoax theory again emerged as the only item that showed a negative association with the feeling of lack of control. For this reason, we excluded the item pertaining to COVID-19 hoax theory from all analyses concerning COVID-19 conspiracy beliefs in the main manuscript both in Study 1 and Study 2.

| **Table S1**. Descriptive statistics for COVID-19 conspiracy belief items and their item-level correlations with news exposure and feelings of anxiety and lack of control in Study 1 | | | | | |
| --- | --- | --- | --- | --- | --- |
|  | *M* (*SD*) | Endorsement rate | News exposure (*r*) | Anxiety (*r*) | Lack of control (*r*) |
| 1. SARS-CoV-2 (coronavirus) is a biological weapon created to eliminate the overcrowded human population | 2.66 (1.39) | 29.8% | –.04 | **.10** | **.21** |
| 2. The state took all the face masks and respirators so that it could give them away to the select few | 2.10 (1.24) | 15.2% | –.05 | **.09** | **.18** |
| 3. The USA is using the SARS-CoV-2 (coronavirus) to settle its conflicts with China | 2.48 (1.30) | 21.4% | –.07 | .05 | **.13** |
| 4. SARS-CoV-2 (coronavirus) was created by the USA to disrupt the European Union so that they could subsequently “save it” | 2.29 (1.30) | 18.4% | **–.09** | .01 | **.11** |
| 5. COVID-19 (coronavirus) epidemic could have been stopped right at the start, but the large companies made a business out of keeping it going | 2.84 (1.31) | 32.6% | –.04 | **.14** | **.20** |
| 6. COVID-19 (coronavirus) is only a fabrication, it is an ordinary flu that pharmaceutical companies rebranded to increase the sales of drugs | 2.15 (1.23) | 15.2% | **–.17** | **–.12** | **–.13** |
| 7. The governments are concealing the real numbers of fatalities especially in children and young people, to prevent panic | 2.49 (1.31) | 23.6% | .04 | **.19** | **.35** |
| 8. SARS-CoV-2 (coronavirus) is artificially created to increase people’s dependency on the pharmaceutical business | 2.59 (1.39) | 28.2% | **–.09** | **.10** | **.11** |
| 9. China is responsible for the spread of the SARS-CoV-2 (coronavirus) – it is its revenge for the protests in Hong Kong | 1.97 (1.06) | 7.2% | .02 | **.15** | **.20** |
| 10. The Chinese government already has the cure for COVID-19 (coronavirus) but they are keeping it secret | 2.22 (1.15) | 12.4% | .05 | **.15** | **.24** |
| 11. The Chinese created SARS-CoV-2 (coronavirus) as a biological weapon which then got out of hand. | 2.28 (1.17) | 14.2% | .04 | **.19** | **.24** |
| 12. The Chinese government intentionally sells ineffective face masks and dubious tests for the coronavirus to Europe to make the course of the pandemic worse in our countries. | 1.83 (0.99) | 5.2% | .01 | **.18** | **.23** |
| *Note*. The table shows means, standard deviations, and endorsement rates (the percentage of the sample which answered with “agree” or “strongly agree”) of the particular conspiracy theory items, as well as their correlations with news exposure, and feelings of anxiety and lack of control. Correlations are based on 501 observations. Significant correlations (*p* < .05) are presented in bold. | | | | | |

| **Table S2**. Descriptive statistics for COVID-19 conspiracy belief items and their item-level correlations with news exposure and feelings of anxiety and lack of control in Study 2 | | | | | |
| --- | --- | --- | --- | --- | --- |
|  | *M* (*SD*) | Endorsement rate | News exposure (*r*) | Anxiety (*r*) | Lack of control (*r*) |
| 1. SARS-CoV-2 (coronavirus) is a biological weapon created to eliminate the overcrowded human population. | 2.79 (1.44) | 32.6% | **–.18** | .05 | .04 |
| 2. COVID-19 (coronavirus) epidemic could have been stopped right at the start, but the large companies made a business out of keeping it going. | 2.87 (1.43) | 34.7% | **–.23** | .04 | .00 |
| 3. COVID-19 (coronavirus) is only a fabrication, it is an ordinary flu but the government is intentionally using it to restrict our civil rights. | 2.20 (1.39) | 20.2% | **–.28** | –.01 | **–.09** |
| 4. COVID-19 pandemic was artificially incited in order to spread fear and subsequently control the population. | 2.58 (1.49) | 29.6% | **–.26** | .05 | –.05 |
| 5. The governments are concealing the real numbers of fatalities especially in children and young people, to prevent panic. | 2.22 (1.32) | 17.4% | **–.08** | .06 | **.15** |
| 6. Bill Gates sponsors vaccines against COVID-19 so that he can spread nano-chips into people through them. | 2.00 (1.28) | 13.3% | **–.17** | –.01 | .01 |
| 7. Governments want to use compulsory vaccination against COVID-19 to achieve monitoring of the population. | 2.28 (1.43) | 22.3% | **–.20** | .02 | –.04 |
| 8. Widespread testing is a way for the government to obtain the DNA of the population so that it can subsequently harbor it and abuse it. | 1.87 (1.22) | 11.6% | **–.19** | .05 | .00 |
| 9. Reporters, scientists, and government all take part in concealing important information about the association between COVID-19 and 5G network. | 2.22 (1.36) | 18.8% | **–.17** | .03 | .03 |
| 10. COVID-19 pandemic is intentionally used for the genocide of domestic population to make a room for migrants to move in. | 2.04 (1.33) | 15.6% | **–.22** | .01 | –.01 |
| *Note*. The table shows means, standard deviations, and endorsement rates (the percentage of the sample which answered with “agree” or “strongly agree”) of the particular conspiracy theory items, as well as their correlations with news exposure, and feelings of anxiety and lack of control. Correlations are based on 1024 observations. Significant correlations (*p* < .05) are presented in bold. | | | | | |

**Section B**

**Comparison of prejudice against Chinese, Italian, and Roma people in Study 1**

From the descriptive statistics presented in Table 1 in the main document, it was clear that the participants exhibited higher negative feelings, social distance, and refusal of help when it came to Roma people, in comparison with Chinese and Italian people. Indeed, as the results of the paired samples t-tests show, that negative emotions toward Roma people were much higher than toward Italian people, *t*(500) = 21.9, *p* < .001, *d* = 0.98, and Chinese people, *t*(500) = 14.4, *p* < .001, *d* = 0.64. Participants also exhibited more negative feelings against Chinese in comparison with Italian people, *t*(500) = 10.8, *p* < .001, *d* = 0.48. The same was found with regard to social distance, which was again much higher in case of Roma people when compared with Italian, *t*(500) = 23.3, *p* < .001, *d* = 1.04, and Chinese people, *t*(500) = 19.6, *p* < .001, *d* = 0.88. Social distance from Chinese people was in turn higher in comparison with Italian people, *t*(500) = 8.34, *p* < .001, *d* = 0.37. Finally, the same pattern of results, albeit less pronounced, was found when considering the refusal of help. Refusal of help to Roma people was higher than against Italian people, *t*(500) = 7.07, *p* < .001, *d* = 0.32, and slightly higher in comparison with Chinese people, *t*(500) = 2.19, *p* = .029, *d* = 0.10. The results also showed higher refusal of help to Chinese in comparison with Italian people, *t*(500) = 6.69, *p* < .001, *d* = 0.30.

**Section C**

**Full-wording of scenarios for the justification of and willingness to engage in violent attacks at 5G masts, non-compliance with government regulations, and violent anti-government protests**

The scenario regarding the violent attacks on 5G network masts was based on materials used by Jolley and Paterson (2020). The other two scenarios were based on real recent events that attracted large media coverage in Slovakia – a series of violent protests against government health-preventive regulations (Slovak Spectator, 2020) and a highly publicized case of a pregnant patient who refused to follow the official safety guidelines, thereby obstructing her admittance to a maternity ward (Šnídl, 2020, October 25). After each scenario, participants were asked how justified are the mentioned actions in their view and how willing they would be to engage in similar actions in the future. The full wording of the scenarios and questions appears below.

***Violent attacks at 5G masts:***

In Great Britain, there is an extensive investigation underway regarding arsons at mobile network masts associated with the conspiracy theory that links COVID-19 with the spread of the 5G network.

At several places across Great Britain, people set the mobile towers ablaze as a form of protest.

To what extent do you consider the described behavior to be justified?

1 („*completely unjustified*“) - 7 („*completely justified*“)

Should a 5G network mast be built in your neighborhood in a near future, would you consider participating in a similar form of protest against the installation of 5G networks as the one described above in news from Great Britain?

1 („*definitely not*“) - 7 („*definitely yes*“)

***Non-compliance with government regulations:***

On October 23^rd^ 2020, Košice University hospital of L. Pasteur admitted a patient to the gynecological-maternity ward who was in an advanced state of pregnancy. According to the hospital, the preparations for childbirth were complicated by the fact that the patient refused to follow the doctor’s instructions regarding the COVID-19 preventive measures: the patient refused to get a test for COVID-19 and to wear a face-mask.

To what extent do you consider the described behavior of the patent to be justified?

1 („*completely unjustified*“) - 7 („*completely justified*“)

Should you have to undergo surgery at a hospital in a near future, would you be willing to follow the preventive measures against the spread of COVID-19 (getting a test for COVID-19, wearing a face-mask)?

1 („*definitely not*“) - 7 („*definitely yes*“)*

* The responses to this question were recoded in a way so that the higher score indicated a lower willingness to follow the preventive measures.

***Violent anti-government regulations protest:***

On October 17^th^ 2020, a protest against government regulations related to COVID-19 took place in front of the Government Office in Bratislava. At the location, 400 to 500 people met under the surveillance of police officers. Protesters managed to throw flares at the Government Office complex, some of the participants of the protest even started to throw stones and bottles at the police.

To what extent do you consider the described behavior to be justified?

1 („*completely unjustified*“) - 7 („*completely justified*“)

Would you consider attending a similar form of protest in the near future against government regulations related to COVID-19?

1 („*definitely not*“) - 7 („*definitely yes*“)

**Section D**

**Regression models for the negative social outcomes associated with the endorsement of generic (not COVID-19 related) conspiracy theories in Study 2**

The regression analyses in Study 2 of the main manuscript showed the predictors of justification of and willingness to engage in violent actions and non-compliance with regulations. However, as was obvious from the correlation analysis (Table 5 in the main manuscript), generic conspiracy theory beliefs (not related to COVID-19 pandemic) showed almost the same patterns of correlations with negative social outcomes as did the COVID-19 conspiracy beliefs. This is not surprising, as people who believe some conspiracy theories tend to also believe other, non-related, even contradictory conspiracy narratives both regarding the COVID-19 pandemic (Miller, 2020) and in more general (Swami et al., 2011). Consistently with this, COVID-19 conspiracy theory beliefs were shown to be strongly correlated with generic conspiracy beliefs in Study 2 presented in the main manuscript (*r* = .75, *p* < .001) as well as in previous research (e.g. Šrol et al., 2021). While people who endorse conspiracy theories about the COVID-19 pandemic likely endorse other conspiracy theories as well, we wanted to see whether the patterns of results would differ between those two types of conspiracy theory beliefs. We have, therefore, rerun the three regression models presented in Table 6 in the main manuscript with generic conspiracy theory beliefs as a predictor. The results are presented in Table S3.

As can be seen from the table below, the results are almost identical to the ones presented in the main manuscript, albeit the generic COVID-19 conspiracy belief endorsement is somewhat more weakly predictive of justification and willingness to engage in government regulations non-compliance, 5G violence, and violent anti-government protests in comparison with COVID-19 conspiracy belief endorsement. The remarkable similarity in the results is likely because negative social outcomes under examination here are not specific only to COVID-19 conspiracy theories. People who believe various conspiracy theories, or have a higher conspiracy mentality, were previously found to have lower trust in institutions, such as one’s government (Šrol et al., 2021), as well as higher anger and willingness for general violence (Jolley & Paterson, 2020). The analyses below show that generic conspiracy theory beliefs predict, along with lower trust in government regulations and/or higher anger and hostility, also justification and willingness to engage in specific forms of violent protests (arsons at 5G masts, violent anti-government protests) as well as non-compliance with government issued regulations.

| **Table S3.** The results of hierarchical linear regression predicting negative social outcomes | | | | | | |
| --- | --- | --- | --- | --- | --- | --- |
|  | 5G violence justification and willingness | | Regulations non-compliance justification and willingness | | Violent protest justification and willingness | |
| **Predictor** | *β* | 95% CI | *β* | 95% CI | *β* | 95% CI |
| *Step 1* | *ΔR^2^ = 0.051* | | *ΔR^2^ = 0.039* | | *ΔR^2^ = 0.045* | |
| Age | **.06** | **[0.01, 0.11]** | –.02 | [–0.07, 0.02] | .03 | [**–**0.02, 0.08] |
| Gender | **.07** | **[0.02, 0.13]** | –.03 | [–0.08, 0.01] | –.05 | [–0.10, 0.00] |
| Education | **–.09** | **[–0.15, –0.04]** | **–.06** | **[–0.11, –0.02]** | **–.07** | **[–0.13, –0.02]** |
| *Step 2* | *ΔR^2^ = 0.219* | | *ΔR^2^* = 0.364 | | *ΔR^2^ = 0.317* | |
| Generic conspiracy beliefs | **.47** | **[0.42, 0.53]** | **.24** | **[0.19, 0.30]** | **.24** | **[0.18, 0.4229** |
| Anger and hostility | .05 | [**–**0.01, 0.10] | –.02 | [**–**0.07, 0.03] | **.10** | **[0.05, 0.15]** |
| Trust in regulations | **–** | **–** | **–.48** | **[–0.53, –0.43]** | **–.41** | **[–0.46, –0.35]** |
| *Full model* | *R^2^ = 0.270* | | *R^2^ = 0.403* | | *R^2^ = 0.362* | |
| *Note*. The table shows the results of three hierarchical linear regressions predicting negative social outcomes with several demographic predictors (Step 1) and variables related to the endorsement of conspiracy beliefs (Step 2). Standardized regression coefficients (*β*’s) and their 95% confidence intervals are presented for every predictor in the final model. Also, the table shows the change in *R^2^* for the both steps of the model as well as the final model. Significant predictors (*p* < .05) are presented in bold. Gender was coded as 1 = “men”, 2 = “women”. | | | | | | |

**Section E**

**The comparison of correlations between generic and China-specific COVID-19 conspiracy beliefs and prejudiced views of the three social outgroups in Study 1**

| **Table S4**. Correlations of generic and China-specific COVID-19 conspiracy theories and prejudice toward the three social outgroups | | | |
| --- | --- | --- | --- |
|  | China-specific COVID-19 CTs | Generic COVID-19 CTs | difference in two dependent correlations (two-tailed) |
| China – negative feeling | **.19** | .02 | ***Z* = 4.47, *p* < .001** |
| China – social distance | **.30** | **.21** | ***Z* = 2.40, *p* = .016** |
| China – refusal of help | **.18** | .05 | ***Z* = 3.21, *p* = .001** |
| Italy – negative feeling | **.13** | **.09** | *Z* = 0.85, *p* = .395 |
| Italy – social distance | **.24** | **.18** | *Z* = 1.71, *p* = .087 |
| Italy – refusal of help | **.17** | **.16** | *Z* = 0.16, *p* = .873 |
| Roma – negative feeling | **.11** | **.20** | ***Z* = –2.45, *p* = .014** |
| Roma – social distance | **.19** | **.20** | *Z* = –0.18, *p* = .857 |
| Roma – refusal of help | **.18** | **.30** | ***Z* = –3.11, *p* = .002** |
| *Note*. Correlations are based on 501 observations. Significant correlations (*p* < .05) are presented in bold. The difference test between two dependent correlation coefficients was conducted in R package *psych*, based on calculations outlined by Steiger (1980). | | | |

**Supplementary references**

Imhoff, R., & Lamberty, P. (2020). A bioweapon or a hoax? The link between distinct conspiracy beliefs about the Coronavirus disease (COVID-19) outbreak and pandemic behavior. *Social Psychological and Personality Science*, *11*(8), 1110-1118. https://doi.org/10.1177/1948550620934692

Jolley, D., & Paterson, J. L. (2020). Pylons ablaze: Examining the role of 5G COVID-19 conspiracy beliefs and support for violence. *British Journal of Social Psychology*, *59*(3), 628–640. https://doi.org/10.1111/bjso.12394

Miller, J. M. (2020). Do covid-19 conspiracy theory beliefs form a monological belief system? *Canadian Journal of Political Science*, *53*(2), 319–326. https://doi.org/10.1017/S0008423920000517

Slovak Spectator (2020). *Unauthorised protest against COVID-19 measures turn violent in Bratislava.* https://spectator.sme.sk/c/22512977/unauthorised-protest-against-covid-19-measures-turns-violent-in-bratislava.html

Šnídl, V. (2020, October 25). *Žena v pôrodnici odmietala rúško aj test na covid. Na diaľku ju povzbudzovala advokátka*. https://dennikn.sk/2106737/zena-v-porodnici-odmietala-rusko-aj-test-na-covid-na-dialku-ju-povzbudzovala-advokatka/

Šrol, J., Mikušková, E. B., & Cavojova, V. (2021). When we are worried, what are we thinking? Anxiety, lack of control, and conspiracy beliefs amidst the COVID-19 pandemic. *Applied Cognitive Psychology*. <https://doi.org/10.1002/acp.3798>

Steiger, J. H. (1980). Tests for comparing elements of a correlation matrix. *Psychological Bulletin*, *87*(2), 245–251. <https://doi.org/10.1037/0033-2909.87.2.245>

Swami, V., Coles, R., Stieger, S., Pietschnig, J., Furnham, A., Rehim, S., & Voracek, M. (2011). Conspiracist ideation in Britain and Austria: Evidence of a monological belief system and associations between individual psychological differences and real-world and fictitious conspiracy theories. *British Journal of Psychology*, *102*(3), 443–463. https://doi.org/10.1111/j.2044-8295.2010.02004.x

van Mulukom, V., Pummerer, L. J., Alper, S., Bai, H. M., Čavojová, V., Farias, J., ... & Žeželj, I. (n.d.) Antecedents and consequences of COVID-19 conspiracy beliefs: a rapid review of the evidence. *PsyArXiv*. https://psyarxiv.com/u8yah/
